# Identification and characteristics of mutations promoting occult HBV infection by ultrasensitive HBsAg assay

**DOI:** 10.1128/jcm.02071-24

**Published:** 2025-03-31

**Authors:** Shi Song, Qian Su, Ying Yan, Huimin Ji, Huizhen Sun, Kaihao Feng, Abudulimutailipu Nuermaimaiti, Shana Halemubieke, Ling Mei, Xinru Liu, Zhuoqun Lu, Le Chang, Lunan Wang

**Affiliations:** 1 National Center for Clinical Laboratories, Institute of Geriatric Medicine, Chinese Academy of Medical Sciences, Beijing Hospital/National Center of Gerontology 665500 , Beijing, China; 2 Beijing Engineering Research Center of Laboratory Medicine, Beijing Hospital 117555, Beijing, China; 3 National Center for Clinical Laboratories, Peking Union Medical College, Chinese Academy of Medical Sciences 12501, Beijing, China; The University of North Carolina at Chapel Hill School of Medicine, Chapel Hill, North Carolina, USA

**Keywords:** occult hepatitis B virus infection, ultrasensitive HBsAg assay, mutation, antigenicity, secretion impairment

## Abstract

The significance of occult hepatitis B virus (HBV) infection (OBI) has been increasingly recognized while the underlying mechanisms remain incompletely understood. This study aimed to identify high-frequency OBI-related mutations in HBV surface antigen (HBsAg)-negative samples tested by the ultrasensitive Lumipulse G HBsAg-Quant assay. OBI samples were collected from 32 blood establishments across 14 provinces in China. Lumipulse G HBsAg-Quant assay was performed for the re-testing and reclassification of OBI. Mutations in genotypes B (GTB) and C (GTC) were analyzed to identify high-frequency single and combined mutations. Additionally, the efficacy of commercial reagents commonly employed in clinical diagnostics for detecting mutant HBsAg was evaluated. Western Blot was used for the confirmation of extracellular HBsAg as well as the detection of intracellular HBsAg. Hydrophilicity analysis and transmembrane distribution prediction of HBsAg were utilized for further validation. Single mutations at 17 sites and 9 combined mutations in GTB indicated a significantly elevated mutation frequency. In GTC, there were single mutations at 16 sites and 9 combined mutations. Several commercial reagents commonly demonstrated limited capacity toward mutant HBsAg with T123A/P, K141C, and P142R/I/K/L (GTB) and S114A/P (GTC). The findings indicated that mutations including T123A/C/K/S, S132G/Y, P142L/R/S/T, T143M, D144G, G145A, K160R+V168A, I4T+V168A, M103I+K122R, and M103I+Q181R (GTB), along with Q101H, M103I, R160K+C221Y (GTC), were associated with reduced levels of HBsAg both extracellularly and intracellularly. Additionally, K160R (GTB) and E2G (GTC) were associated with intracellular aggregation. This study elucidates the mutations associated with decreased extracellular HBsAg with ultrasensitive HBsAg assay, providing insight for further investigation into the mechanisms of OBI.

**IMPORTANCE:**

The sensitivity of HBsAg detection reagents directly impacts the identification of occult hepatitis B virus (HBV) infection (OBI). This study aims to identify high-frequency OBI-related mutations in HBV surface antigen (HBsAg)-negative samples evaluated using a Fujirebio-Lumipulse ultrasensitive HBsAg assay and to investigate the implications of these mutations on the antigenicity of HBsAg, the detection capacities of various HBsAg assays, and the effects on intracellular and extracellular levels of HBsAg. Generally, our study offers a new perspective on OBI-related mutations by ultrasensitive HBsAg assay and lays the groundwork for further research on the OBI mechanism and the enhancement of HBsAg detection reagents.

## INTRODUCTION

Hepatitis B virus (HBV) infection remains a significant global public health challenge, with the global burden of chronic HBV infection estimated at 316 million people in 2019 (1). As a special state of HBV infection, occult HBV infection (OBI) showed extremely low levels of HBV DNA and the lack of detectable serum HBV surface antigen (HBsAg) (2). Therefore, OBI poses a potential threat to the safety of blood transfusions and is associated with severe liver disease. However, it is under-recognized in current hepatitis B guidelines of EASL (3), AASLD (4), and CMA (5). The prevalence of OBI is estimated at approximately 0.09% in the global population (6), and around 0.01% in blood donors from an international study (7). These data underscore the importance of recognizing OBI and the need for mechanistic studies into OBI.

With the increasing popularity of nucleic acid detection and improvements in detection sensitivity, an increasing number of OBIs have been identified (8, 9). Consequently, the detection of serum HBsAg has progressively emerged as a crucial aspect for the diagnosis of OBI. Nevertheless, some studies have demonstrated that the extremely low levels of serum HBsAg in OBI may result in false negativity when using conventional HBsAg assays (10, 11). Furthermore, mutations within the α determinant of the major hydrophilic region (MHR) of HBsAg may impede the effective recognition of antigens by specific antibodies. Meanwhile, the formation of antigen-antibody complexes may also interfere with the detection of HBsAg (12–14). These factors may lead to the missed detection of HBsAg and the misclassification of HBsAg-positive individuals as OBI cases, thereby influencing the treatment strategies. To eliminate these potential interferences, novel detection assays have been developed. These detection assays, known as ultrasensitive HBsAg assays, effectively enhance the sensitivity for detection through methods such as linearization of HBsAg and detection of both the α determinant and the epitope situated within the lipid bilayer. Including Fujirebio-Lumipulse G HBsAg-Quant assay (Fujirebio, Tokyo, Japan) with a limit of detection (LOD) of 0.005 IU/mL (15) and Abbott-ARCHITECT HBsAg Next with a LOD of 0.0052 IU/mL (16). The use of these ultrasensitive HBsAg detection assays facilitates the detection of extremely low concentrations of HBsAg titers.

HBV mutations are considered a significant mechanism underlying the development of OBI (17). In our previous studies, conventional reagents were employed for the detection of HBsAg and the classification of OBI, including subsequent investigations of OBI-related mutations (18–20). However, some studies have reported that 18.2%–61.5% of samples previously classified as negative by conventional assays tested positive for HBsAg by ultrasensitive methods (10, 15, 21, 22), suggesting that both the classification of OBI and the identification of OBI-related mutations in our prior studies may have been inaccurate due to the use of conventional assays for the detection of HBsAg.

Consequently, in this study, we detected the serum HBsAg of HBV-infected individuals utilizing the Fujirebio-Lumipulse G HBsAg-Quant assay due to the accessibility (15). The OBI-related mutations were then analyzed in newly identified HBsAg-negative samples tested by Fujirebio. We further investigated the impact of these mutations on the antigenicity of HBsAg, as well as their effects on both intracellular and extracellular HBsAg levels. Our study provides perspectives on OBI research, emphasizing the need to focus on the implications of HBV mutations and the sensitivity of HBsAg detection techniques.

## MATERIALS AND METHODS

### Study design and participants

**Fig 1:**
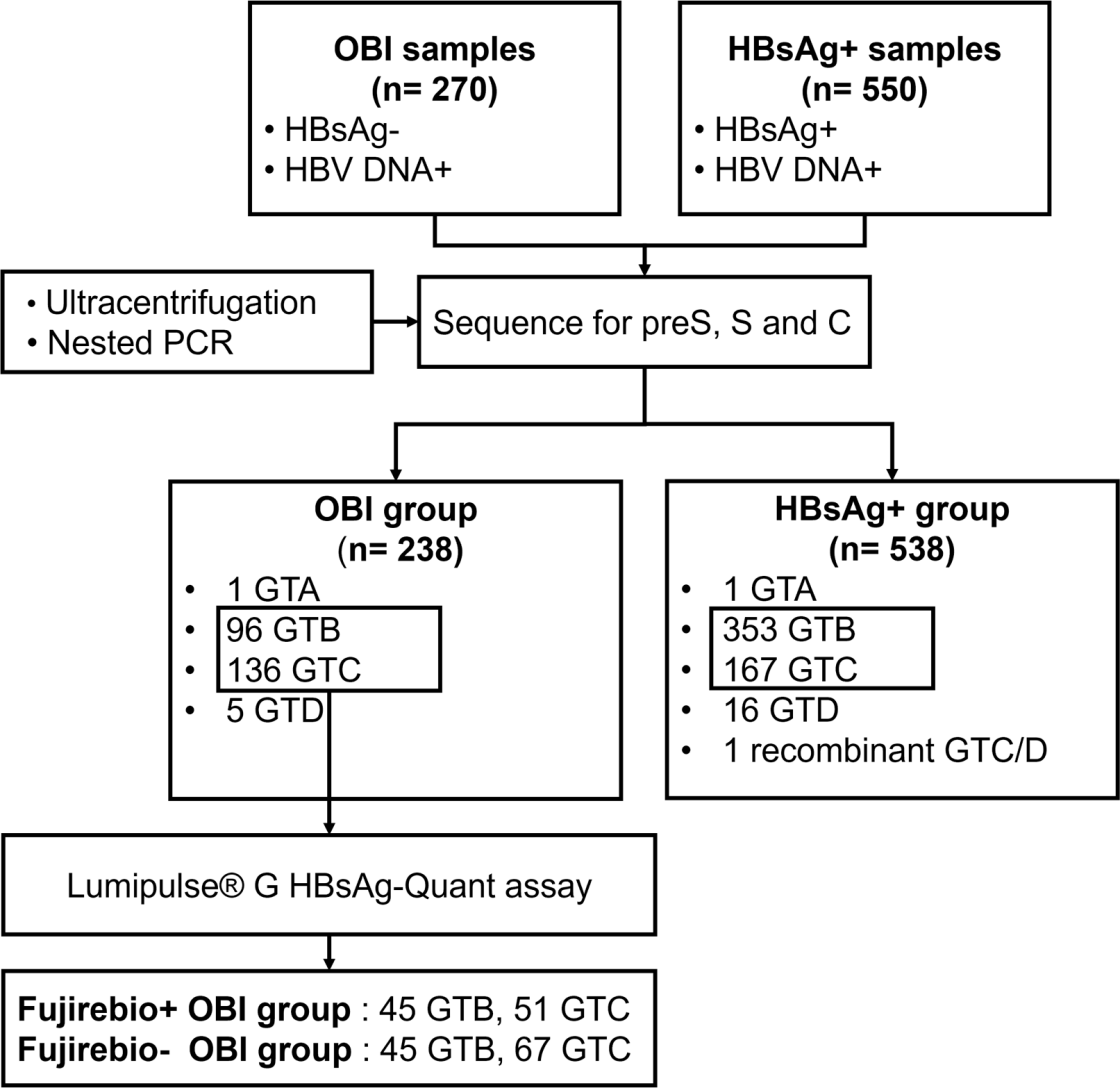
Study design and participants. 270 samples with HBsAg non-reactive and HBV DNA reactive and 550 samples with HBsAg and HBV DNA reactive were collected from 32 blood establishments across 14 provinces. Ultracentrifugation and nested PCR were employed to enhance sequence acquisition. OBI samples were further tested using the Lumipulse G HBsAg-Quant assay and subsequently categorized into Fujirebio+ OBI and Fujirebio− OBI. The distribution of OBI group, Fujirebio− OBI, Fujirebio+ OBI, and HBsAg+ groups are all illustrated in the figure.

We collected and sequenced 270 HBsAg non-reactive and HBV DNA reactive samples and 550 HBsAg reactive and HBV DNA reactive samples from 32 blood establishments across 14 provinces in China. All HBV serological markers were tested using ARCHITECT i2000 (Abbott Laboratories, Chicago, IL, USA), including HBsAg (ARCHITECT HBsAg Qualitative II Reagent Kit, Abbott Ireland Diagnostics Division, Sligo, Ireland; LOD: 0.021 IU/mL), HBV surface antibody (anti-HBs), HBeAg, anti-HBe, and HBV core antibody (anti-HBc). HBV DNA was performed using the Cobas TaqScreen MPX test version 2.0 (Roche Molecular Systems, Inc., NJ, USA; LOD 2.3 IU/mL). OBI samples with successful sequencing in the S region were included in this study for further analysis using the Fujirebio-Lumipulse G HBsAg-Quant assay. The implementation of retest and confirmation reagents—Lumipulse G HBsAg-Quant Confirmation (Fujirebio, Tokyo, Japan) was conducted to enhance the precision of the results. The OBI group was further divided into Fujirebio+ OBI group (positive in Fujirebio-Lumipulse assay) and Fujirebio− OBI groups (negative in Fujirebio− Lumipulse assay), respectively. The OBI group, Fujirebio+ OBI group, Fujirebio− OBI group, and HBsAg+ group are analyzed in this study (Fig. 1).

### Amplification and sequence analysis of the preS, S, and C regions

The viral particles in OBI and HBsAg+ samples were extracted from 1.2 mL plasma. The preS region, S region, and C region of strains were amplified by nested-PCR with Amplitaq DNA polymerase (Applied Biosystems, Carlsbad, CA, USA) and sequenced by Sanger sequencing. To enhance sequencing efficiency, an ultracentrifugation protocol was implemented, wherein 8.0 mL of plasma was subjected to centrifugation at 50,000 rpm (equivalent to 171,200 × *g*) for 4 hours using the Sorvall WX+ ultracentrifuge (Thermo Scientific Inc., Germany). The primers used for nested PCR and sequencing are listed in Table S1. For HBV genotyping, the strains of the S region were compared with the reference sequences of genotypes A, B1-6, C1-16, D, F, G, and H obtained from GenBank. Phylogenetic analysis of the preS, S, and C regions was conducted among the OBI group, Fujirebio− OBI group, Fujirebio+ OBI group, and the HBsAg+ group. This analysis utilized Geneious Prime 8, Mega 7, and the online software Viral Epidemiology Signature Pattern Analysis (VESPA, https://www.hiv.lanl.gov/content/sequence/VESPA/vespa.html) (23).

### Characterization of mutations in the S region

#### Wild-type and mutant full-length HBV genome plasmids

The pGEM-4Z plasmid containing 1.3× genotype B4 HBV genome and the pBlueBac 4.5 plasmid containing 1.2× genotype C2 HBV genome were generously provided by Professor Fengmin Lu from Peking University Health Science Center in China (24). Mutations were generated through site-directed mutagenesis. The wild--type (WT HBV plasmid was amplified with 2× TransStart FastPfu Fly PCR SuperMix (TransGen Biotech, Beijing, China) and mutant primers (Table S2) designed by the QuikChange Primer Design tool (https://www.agilent.com.cn/store/primerDesignProgram.jsp). The template plasmid was then digested with DpnI enzyme (New England Biolabs, MA, USA), and the expected plasmids were isolated later. Combined mutations were subsequently introduced based on the single mutations. The constructed plasmids were confirmed by Sanger sequencing.

#### Cell culture and plasmid transfection

Huh-7 cells (Procella, Wuhan, Hubei, China) were maintained in Dulbecco’s modified Eagle’s medium (Corning, Manassas, VA, USA) supplemented with 10% fetal bovine serum (Gibco, Australia) and 1‰ TransSafe Mycoplasma Prevention Reagent (TransGen Biotech, Beijing, China). Cells were incubated at 37°C with 5% CO_2_ and seeded into 6-well plates before transfection. A total of 2,000 ng WT or mutant HBV plasmids were transfected into Huh-7 cells with 60%–90% confluence using the Lipofectamine 3000 Transfection Reagent (Invitrogen, Carlsbad, CA, USA). 500 ng pSELECT-zeo-SEAP plasmid (InvivoGen, San Diego, CA, USA) was co-transfected to standardize the transfection efficiency. Cells and supernatants were harvested 72 hours after transfection.

#### Quantification of extracellular HBsAg

Culture supernatants were collected 72 hours after transfection and centrifuged to remove the free-cell precipitations. Based on the linear detection range of the reagents, the transfection supernatant was appropriately diluted and then detected by conventional detection assays, including DiaSorin-Murex HBsAg Version 3 (DiaSorin, Saluggia, Italy; LOD: 0.05 IU/mL), Abbott-HBsAg Reagent Kit (Abbott Ireland Diagnostics Division, Sligo, Ireland; LOD: 0.05 IU/mL), and Roche-HBsAg II quant (Roche Diagnostics GmbH, Mannheim, Germany; LOD: 0.05 IU/mL), as well as ultrasensitive assay of Fujirebio-Lumipulse G HBsAg-Quant assay. For the detection of extracellular HBsAg, these reagents were abbreviated using their respective company names as DiaSorin, Abbott, Roche, and Fujirebio, respectively. The supernatants were also tested for Seap activity using the QUANTI-Blue Solution kit (InvivoGen, San Diego, CA, USA). To minimize errors caused by variations in transfection efficiency, the ratio of extracellular HBsAg results to Seap detection results was used as the outcome for each reaction group. Results were expressed as a relative value compared to the WT constructs.

#### Western blot analysis

For intracellular HBsAg, cells were harvested 72 hours after transfection. The cells were washed three times with 1× phosphate-buffered saline (Lablead, Beijing, China) and lysed by NP-40 buffer with 1% PMSF (Solarbio, Beijing, China). The prepared samples were separated by SDS-PAGE and subsequently transferred to a PVDF membrane (Sigma-Aldrich, Shanghai, China). The associated single mutations of combined mutations were also included on the same membrane. HBsAg was detected by the primary horse polyclonal antibody (Abcam, ab9193, Cambridge, UK), with an anti-β tubulin antibody (Abcam, ab6046, Cambridge, UK) serving as a loading control. The densitometry of bands was detected with the Amersham ImageQuant 800 (Cytiva, Tokyo, Japan) and the ImageQuant TL software. Results were expressed as a relative value compared to the WT constructs.

#### Protein hydrophilicity analysis

Phyre2 online analysis software (http://www.sbg.bio.ic.ac.uk/phyre2) was used to predict the tertiary structure of mutant HBsAg. Results were analyzed with MolSTAR protein structure drawing software (https://www.novopro.cn/tools/molstar.html). ExPASy ProtScale online analysis software (https://web.expasy.org/protscale/) was used to analyze the hydrophilic and hydrophobic properties of mutant HBsAg (25). Results were displayed as an amino acid scale and visualized through a line graph.

### Statistics analysis

Descriptive statistics were employed to present the background characteristics. The Shapiro-Wilk test was performed to assess the normality of continuous variables. The Mann-Whitney U test was used to compare the serological markers. The Chi-square test or Fisher’s exact test was used to compare the mutant frequency of preS, S, and C regions. Fisher’s exact test and co-variation analysis were performed on combined mutations in the S region derived from monoclonal sequences. Co-variations with a coefficient of binomial correlation (Phi) >0.3 and *P* < 0.05 were considered statistically significant positive combined mutations. The unpaired two-tailed t-test was used to compare extracellular and intracellular HBsAg among different groups. Statistical analysis was conducted using IBM SPSS software Statistic 22 (SPSS, Chicago, IL, USA) and GraphPad Prism 8 (GraphPad Inc., San Diego, CA, USA).

## RESULTS

### Characteristic and serological analysis of OBI samples

In this study, the S region of virus strains from 238 OBI samples and 538 HBsAg+ samples were successfully sequenced. The evolutionary analysis of the S region identified various genotypes: 1 (0.42%) genotype A, 96 (40.34%) genotype B, 136 (57.14%) genotype C, and 5 (2.10%) genotype D in the OBI group; 1 (0.19%) genotype A, 353 (65.61%) genotype B, 167 (31.04%) genotype C, 16 (2.97%) genotype D, and 1 (0.19%) recombinant genotype C/D samples in the HBsAg+ group (Fig. 1). Given the predominance of genotypes B and C, which are common in China, subsequent analyses focused on these two genotypes. Successfully obtained sequences in the preS region, S region, and C region of genotype B and C in the OBI group and HBsAg+ group were provided in Table S3. Excluding samples with insufficient serum volume, HBsAg was identified as positive using the Fujirebio-Lumipulse assay in 50.00% (45/90) genotype B and 43.22% (51/118) genotype C OBI samples (Fig. 1).

**Fig 2:**
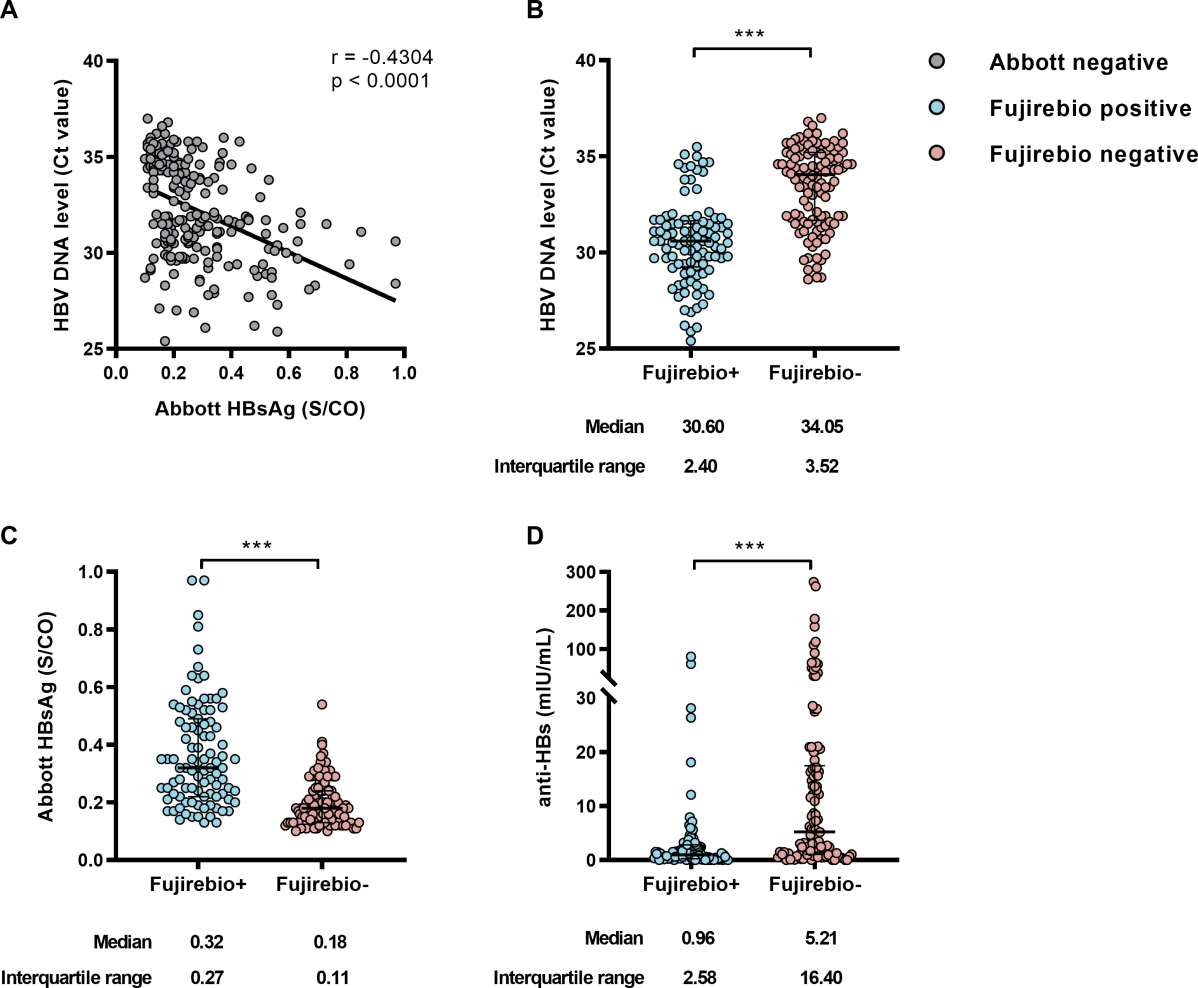
Serological characteristics of OBI, Fujirebio+ OBI, and Fujirebio− OBI groups. The Abbott HBsAg, Fujirebio HBsAg, and HBV DNA were detected by HBsAg Qualitative II Reagent Kit, Lumipulse G HBsAg-Quant assay, and Roche TaqScreen MPX version 2, respectively. (**A**) A notable correlation was identified between the Abbott HBsAg level (S/CO) and HBV DNA level (CT values), r = −0.4304, *P* < 0.0001. Serum level of HBV DNA (**B**), Abbott HBsAg (**C**), and anti-HBs (D) levels between the Fujirebio+ and Fujirebio− OBI samples were analyzed. Fujirebio− OBI group obtained a lower HBV DNA level (*P* < 0.001), lower Abbott HBsAg level (*P* < 0.001), and higher anti-HBs level (*P* < 0.001). The corresponding median, interquartile range between Fujirebio+ and Fujirebio− OBI groups were shown. Results were analyzed by normality test and Mann-Whitney U test, *P < 0.05, **P < 0.01, ***P < 0.001.

In the OBI group, the qualitative Abbott HBsAg levels were negatively correlated with HBV DNA Ct values (r = −0.4304, *P* < 0.0001) (Fig. 2A). This was also consistent with the significantly lower HBV DNA Ct values in Fujirebio+ OBI than those in the Fujirebio− OBI group (median 30.6 vs 34.1, *P* < 0.001) (Fig. 2B). Additionally, Fujirebio+ OBI samples exhibited significantly higher levels of signal to cutoff (S/CO) values of HBsAg (median 0.32 vs 0.18, *P* < 0.001) using Abbott-HBsAg Qualitative II Reagent Kit (Fig. 2C), and lower levels of anti-HBs (median 0.96 vs 5.21, *P* < 0.001) (Fig. 2D). The same results were obtained in a separate analysis of genotype B and C samples (Fig. S1A through F). These findings highlight distinct serological characteristics between the Fujirebio+ OBI and Fujirebio− OBI groups.

### Mutations in preS, S, and C regions

**Fig 3:**
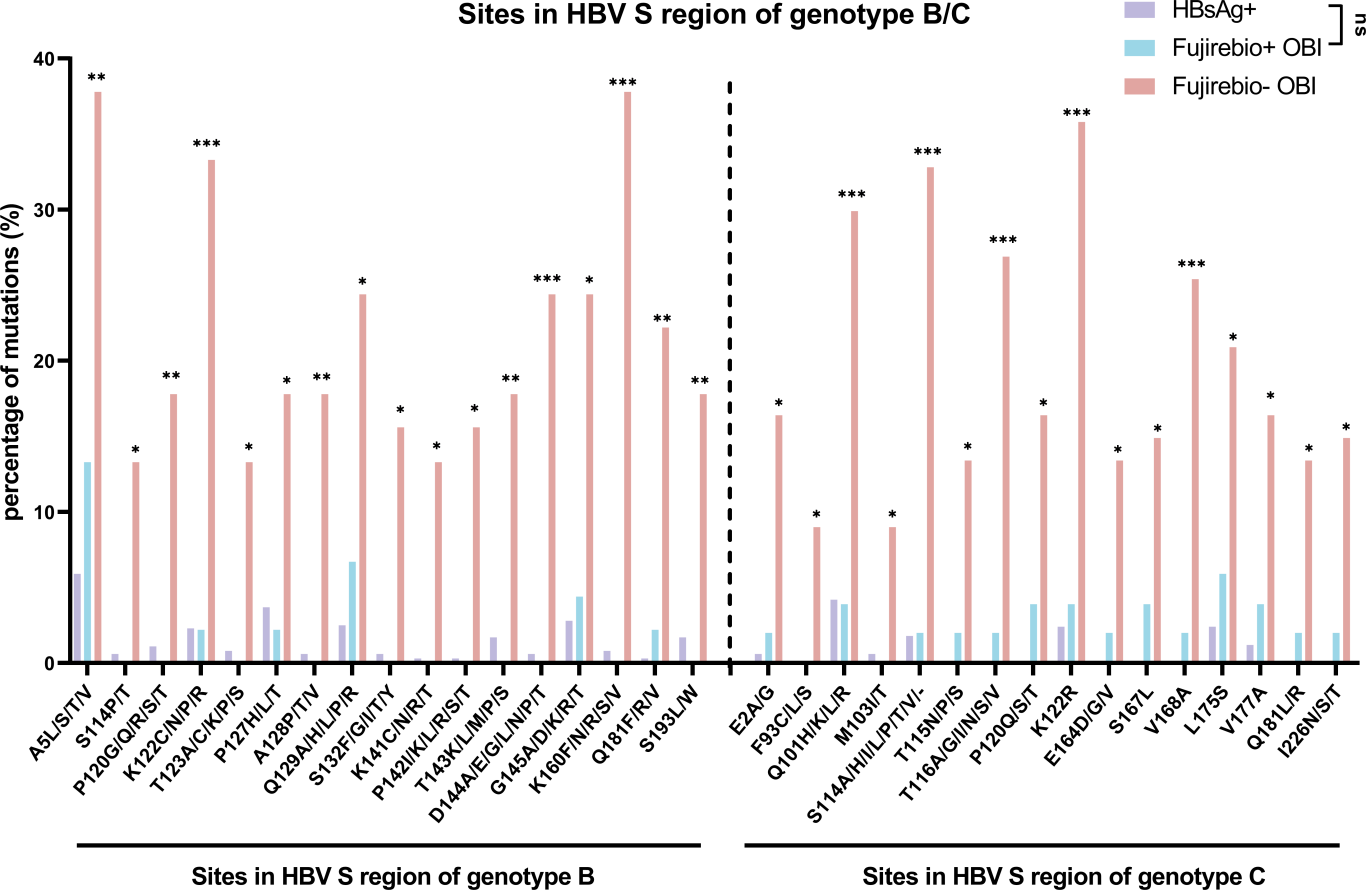
The frequency of mutations at different amino acid sites in the S region significantly elevated in the Fujirebio− OBI group compared to the HBsAg+ group and Fujirebio+ OBI group. In genotype B, N_HBsAg+_ = 353, N_Fujirebio+ OBI_ = 45, N_Fujirebio− OBI_ = 45. In genotype C, N_HBsAg+_ = 165, N_Fujirebio+ OBI_ = 51, N_Fujirebio− OBI_ = 67. Mutations were shown in the format of wild amino acids—sites-mutant amino acids. Statistical analysis was performed using the Chi-square test or the Fisher’s exact test, *P < 0.05, **P < 0.01, ***P < 0.001.

A total of 46.15% (96/208) of OBI samples from genotypes B and C exhibited positivity for HBsAg as determined by the Fujirebio-Lumipulse assay. We observed that the mutation frequencies at some sites in the S region were significantly higher in the OBI group compared to the HBsAg+ group both in genotype B and genotype C (Fig. S2A and D). However, there was no significant difference in the mutation frequencies of the preS and C regions between the HBsAg+ group and the OBI+ group (Fig. S2B, C, E, and F). To investigate the true OBI-related mutations, we concentrated on those that were significantly more frequent in the Fujirebio− OBI group compared to both the Fujirebio+ OBI group and the HBsAg+ group (Fig. 3). These mutations were predominantly observed in the S region of the HBV genome.

Our analysis revealed two distinct patterns of amino acid mutations exhibiting significantly elevated frequencies. A notable observation was the strong residue preference at specific sites within the HBV S region, where mutations frequently converged toward one or two predominant amino acid substitutions. These dominant variants represent the most prevalent site-specific alterations, highlighting their potential biological significance and necessitating focused investigation. In genotype B, these mutations included A5T/S, S114T, P120Q, K122R, P127T, A128V, Q129R, T143M, D144G, G145A, K160R/N, Q181R, and S193L. For genotype C, the mutations were E2G, Q101K/H, M103I, S114A/P, T115N, T116A/S, P120Q/T, K122R, E164G, S167L, V168A, L175S, V177A, Q181R, and I226S. Despite observing statistically significant increases in site-specific mutation frequencies, the amino acid substitution patterns at these sites exhibited considerable heterogeneity, lacking discernible trends in residue preference. To elucidate the potential functional implications of these mutations, we conducted a comprehensive analysis of all amino acid variants occurring at the identified positions. The mutations included in this category were T123A/C/K/P/S, S132F/G/I/T/Y, K141C/N/R/T, and P142I/K/L/R/S/T in genotype B; and F93C/L/S in genotype C.

Besides, naturally existing HBV strains seldom exhibit only single mutations, and the presence of certain combined mutations may better reflect the true situation of various HBV mutations existing in the infected individuals. Consequently, our investigation was expanded to screen for combined mutations within the S region, as these combinations may have a more substantial impact on HBsAg expression and configuration. A significantly higher frequency of prevalent combined mutations was observed in the Fujirebio− OBI group in contrast to the HBsAg+ group. Specifically, nine combined mutations in genotype B and nine combined mutations in genotype C were selected for further analysis (Table 1), along with the corresponding single mutations.

**TABLE 1:** Selection of combined mutations in the S region

Combined mutations	Frequency in Fujirebio− OBI	Frequency in HBsAg+ group	Relationship between mutations^*a*^	
			Phi	*P* value
Genotype B	*n* = 45	*n* = 353		
S174*N*+L175S	9	0	0.321	0.031
K160R+V168A	7	0	0.445	0.005
V168A+P217L	6	2	0.383	0.017
I4T+V168A	6	0	0.383	0.017
K160R+L175S	6	0	0.325	0.050
M103I+Q181R	6	0	0.547	0.001
M103I+K122R	6	0	0.359	0.044
K122R+K160R	5	0	0.327	0.043
V177A+Q181R	5	0	0.412	0.014
Genotype C	*n* = 67	*n* = 165		
K122R+S174N	13	0	0.368	0.003
V168A+S174N	11	0	0.479	0.001
K122R+V168A	11	0	0.351	0.004
R160K+S174N	10	0	0.320	0.009
T118K+S174N	9	0	0.440	0.001
R160K+V168A	9	0	0.310	0.011
V168A+L175S	8	0	0.375	0.002
Q129*P*+V168A	7	0	0.526	<0.001
R160K+C221Y	7	0	0.321	0.009

^*a*^ Co-variations with coefficient of binomial correlation (Phi) > 0.3 and *P* < 0.05 were considered as statistically significant positive combined mutations.

### Functional analysis of the impact of mutations on extracellular HBsAg by various detection techniques

**Fig 4:**
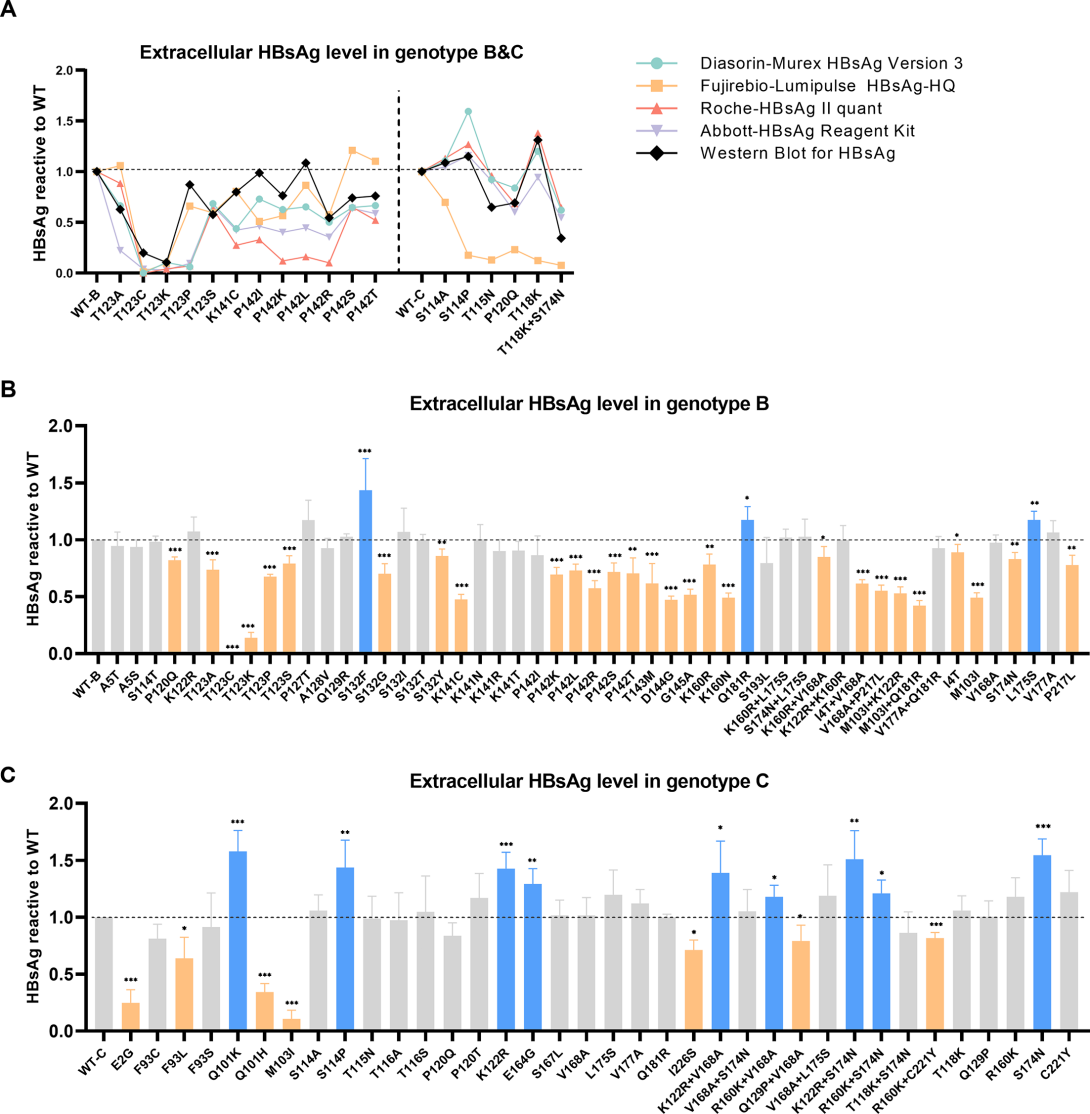
Extracellular HBsAg analysis. The supernatants were collected 72 hours after transfection with WT or mutant plasmids. (**A**) Mutations that show different results were detected by DiaSorin, Abbott, Roche, and Fujirebio. Western blot was conducted for confirmation and shown as a densitometric analysis of small S protein bands. Results were expressed as a relative value compared to the corresponding WT. Results of different methods above were shown in different colors. (**B and C**) Extracellular HBsAg levels (normalized by Seap activity) of mutations in genotype B/C were detected by DiaSorin (T123C/P in genotype B detected by Fujirebio). The mutant samples were displayed as single mutations, combined mutations, and complementary mutations for combined mutations. Results were demonstrated as means and standard deviations from at least three independent experiments and expressed as relative values compared to the WT. Mutations with significantly increased or decreased HBsAg levels were marked in blue or orange, respectively. Statistical analysis was performed using the student’s t-test, *P < 0.05, **P < 0.01, ***P < 0.001.

Previous studies have indicated that mutations such as G145R within the MHR can influence the antigenicity of HBsAg (12, 13). In this study, we evaluated the impact of these mutations on the HBsAg antigenicity and the detection capacities by Fujirebio-Lumipulse assay and various commercial reagents commonly used in clinic practice, including DiaSorin, Abbott, and Roche. While most mutations produced consistent results across these methods, certain mutations led to inconsistent outcomes. We observed that some assays exhibited reduced detection capability for several mutant HBsAg (Fig. S3A and B). Among these mutations, T123C causes a highly significant reduction in HBsAg levels, even resulting in negative detection by the DiaSorin assay. Western blot analysis for supernatant revealed reduced detection sensitivity for mutations such as genotypes B (GTB)-T123C/P with DiaSorin, GTB-T123A/P, P142I/K/L with Abbott, GTB-T123P, K141C, P142I/K/L/R with Roche, and genotypes C (GTC)-S114A/P, T115N, P120Q, T118K, T118K+S174N with Fujirebio (Fig. S3C; Fig. 4A).

Based on the comparison of several detection assays above, the DiaSorin assay demonstrated the most robust detection capability, except for interference caused by the T123C/P mutations. Therefore, we combined the use of Fujirebio to detect GTB-T123C/P and DiaSorin to detect the remaining mutations for subsequent testing. In genotype B, single mutations P120Q, T123A/C/K/P/S, S132G/Y, K141C, P142K/L/R/S/T, T143M, D144G, G145A, K160R/N, and combined mutations K160R+V168A, I4T+V168A, V168A+P217L, M103I+K122R, M103I+Q181R were associated with decreased extracellular HBsAg, whereas S132F and Q181R were linked to elevated extracellular HBsAg (Fig. 4B). In particular, T123C exhibited a notably low concentration that posed challenges for detection. In genotype C, single mutations E2G, F93L, Q101H, M103I, I226S, and combined mutations Q129P+V168A, R160K+C221Y were correlated with reduced extracellular HBsAg levels, while single mutations Q101K, S114P, K122R, E164G, and combined mutations K122R+V168A, R160K+V168A, K122R+S174N, R160K+S174N were associated with increased extracellular HBsAg levels (Fig. 4C). The results of most combined mutations were consistent with those of single mutations. However, cases such as Q129P+V168A and R160K+C221Y in genotype C exhibited reduced extracellular HBsAg levels, while no single mutations lead to decreased HBsAg, indicating that the combined mutations may exert an additional influence on HBsAg expression (Fig. 4C).

### Functional analysis of the impact of mutations on intracellular HBsAg

**Fig 5:**
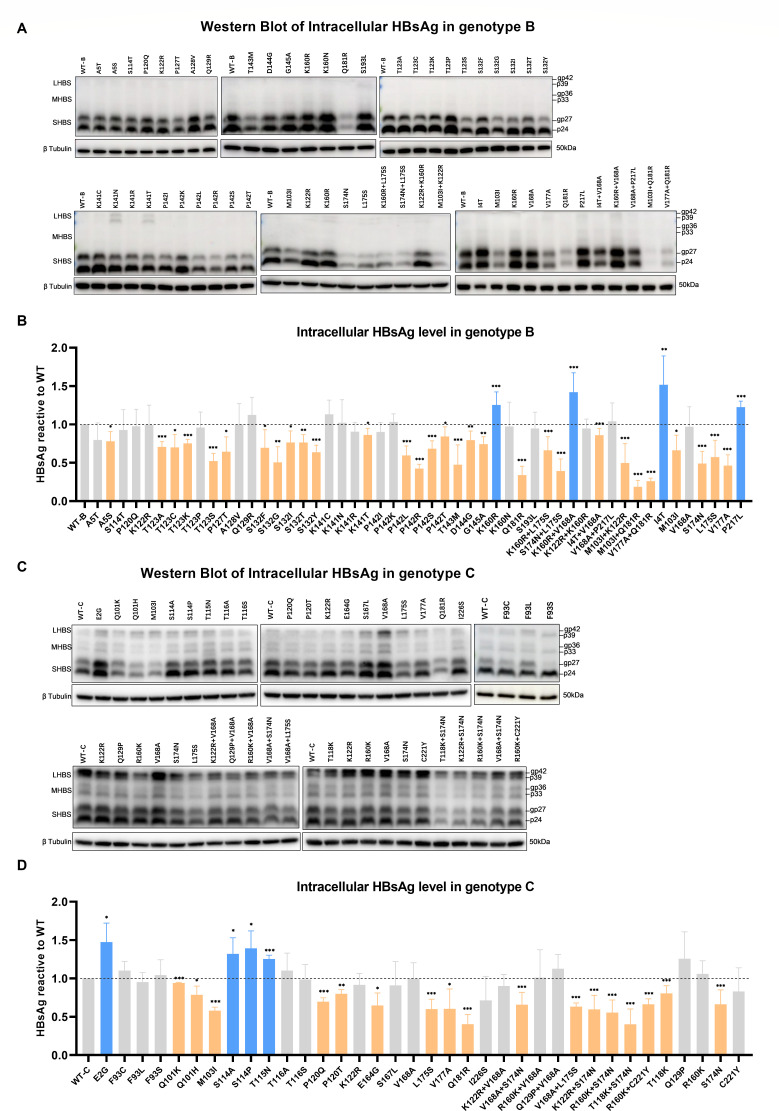
Intracellular HBsAg analysis. The cells were collected 72 hours after transfection with WT or mutant plasmids. The mutant samples were displayed as single mutations, combined mutations, and complementary mutations for combined mutations. (**A**) Western blot of intracellular HBsAg of mutations in genotype B. (**B**) Densitometric analysis of SHBS bands in genotype B. (**C**) Western blot of intracellular HBsAg of mutations in genotype C. (**D**) Densitometric analysis of SHBS bands in genotype C. Densitometric results of SHBS were normalized by β tubulin and expressed as a relative value compared to the WT. Results were demonstrated as means and standard deviations from at least three independent experiments. Mutations with significantly increased or decreased HBsAg levels were marked in blue or orange, respectively. Statistical analysis was performed using the student’s t-test, *P < 0.05, **P < 0.01, ***P < 0.001.

To further corroborate the alterations in extracellular HBsAg, we evaluated the levels of intracellular HBsAg. In genotype B, single mutations A5S, T123A/C/K/S, P127T, S132F/G/I/T/Y, K141T, P142L/R/S/T, T143M, D144G, G145A, Q181R, and combined mutations K160R+L175S, S174N+L175S, I4T+V168A, M103I+K122R, M103I+Q181R, V177A+Q181R were associated with decreased intracellular HBsAg levels. Conversely, the mutations K160R and K160R+V168A were associated with increased extracellular HBsAg levels (Fig. 5A and B). In genotype C, single mutations Q101K/H, M103I, P120Q/T, E164G, L175S, V177A, Q181R, along with the combined mutations V168A+S174N, V168A+L175S, K122R+S174N, R160K+S174N, T118K+S174N, and R160K+C221Y, were associated with reduced intracellular HBsAg levels. Conversely, the mutations E2G, S114A/P, and T115N showed increased extracellular HBsAg (Fig. 5C and D). In the context of combined mutations, I4T+V168A in genotype B (Fig. 5A and B) and R160K+C221Y in genotype C (Fig. 5C and D) resulted in decreased intracellular HBsAg levels, with no single mutation causing a decrease in intracellular HBsAg.

### Analysis on the mechanism and secretion disorder of OBI-related mutation

The findings pertaining to mutations significantly influencing extracellular HBsAg were summarized in Table 2. Certain mutations were associated with a notable reduction in both extracellular and intracellular HBsAg, including T123A/C/K/S, S132G/Y, P142L/R/S/T, T143M, D144G, G145A, I4T+V168A, M103I+K122R, M103I+Q181R in genotype B, and Q101H, M103I, R160K+C221Y in genotype C. These mutations are likely to result in decreased expression levels or diminished stability of HBsAg. Among these mutations, we also observed some mutations, including T123C/K and D144G in genotype B and Q101H and M103I in genotype C, which caused a significant and large proportion decrease in extracellular HBsAg while a slight decrease in intracellular HBsAg. This indicates a reduction in the ratio of extracellular to intracellular HBsAg. These mutations may cause both decreased HBsAg levels and impaired secretion.

**TABLE 2:** Summary of the effect of mutations that result in significant alterations in extracellular HBsAg

Mutations	Extracellular HBsAg^*a*^	Intracellular HBsAg^*a*^	Potential underlying mechanisms	
			Decreased expression or diminished stability	Secretion disorder
Genotype B				
P120Q	↓	–		
T123A	↓	↓	√	
T123C	↓↓↓	↓	√	√
T123K	↓↓↓	↓	√	√
T123P	↓	–		
T123S	↓	↓	√	
S132F	↑	↓		
S132G	↓	↓	√	
S132Y	↓	↓	√	
K141C	↓↓	–		
P142K	↓	–		
P142L	↓	↓	√	
P142R	↓	↓↓	√	
P142S	↓	↓	√	
P142T	↓	↓	√	
T143M	↓	↓↓	√	
D144G	↓↓	↓	√	√
G145A	↓	↓	√	
K160R	↓	↑		√
K160N	↓↓	–		
Q181R	↑	↓↓		
K160R+V168A	↓	↑		√
I4T+V168A	↓	↓	√	
V168A+P217L	↓	–		
M103I+K122R	↓	↓↓	√	
M103I+Q181R	↓↓	↓↓↓	√	
Genotype C				
E2G	↓↓↓	↑		√
F93L	↓	–		
Q101K	↑↑	↓		
Q101H	↓↓	↓	√	√
M103I	↓↓↓	↓	√	√
S114P	↑	↑		
K122R	↑	–		
E164G	↑	↓		
I226S	↓	–		
K122R+V168A	↑	–		
R160K+V168A	↑	–		
Q129P+V168A	↓	–		
K122R+S174N	↑↑	↓		
R160K+S174N	↑	↓		
R160K+C221Y	↓	↓	√	

^*a*^ Mean values of extracellular and intracellular HBsAg were shown as –, no significant change; ↓, ↓↓, and ↓↓↓, significantly decrease (decrease to 50%–100%, 25%–50%, and <25% of WT, respectively); ↑ and ↑↑, significantly increase (increase to 100%–150% and 150%–175% of WT, respectively).

**Fig 6:**
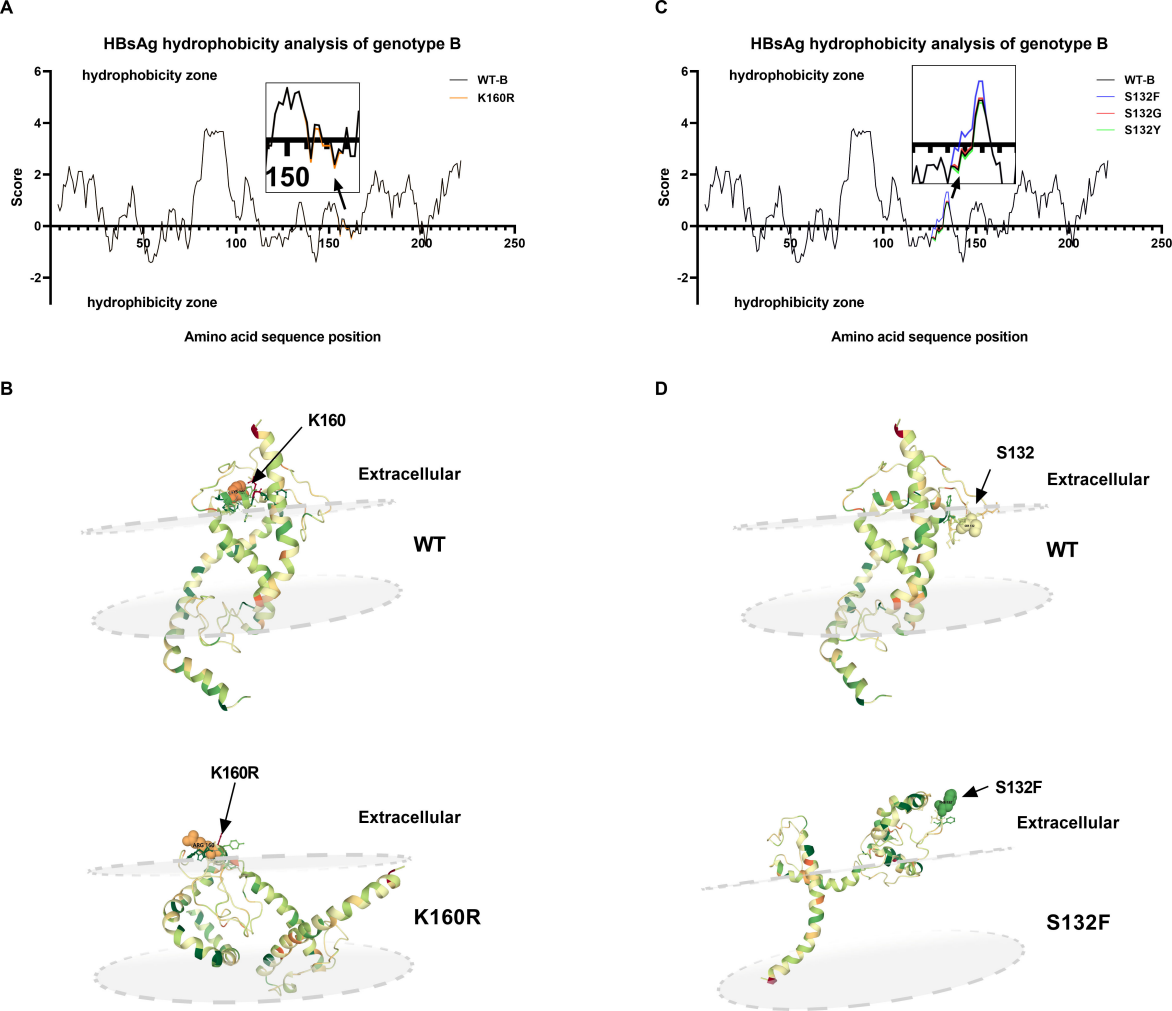
Transmembrane distribution and hydrophobicity analysis of HBsAg. (**A**) Hydrophilicity analysis of WT and K160R. Data were calculated by ExPASy ProtScale. The black line refers to the WT and the orange line refers to the K160R. (**B**) The HBsAg structure and transmembrane distribution prediction of WT and K160R. The focused locations and mutations were marked with black arrows. (**C**) Hydrophilic and hydrophobic analysis of WT and S132F/G/Y. The results of S132F/G/Y were shown with blue, red, and green lines, respectively. (**D**) The HBsAg structure and transmembrane distribution prediction of WT and S132F.

Notably, the presence of K160R in genotype B and E2G in genotype C led to a decrease in extracellular HBsAg and an increase in intracellular HBsAg. The K160R, located at the boundary of the MHR and transmembrane domain (TMD) three was found to disrupt hydrophilicity (Fig. 6A). Besides, the transmembrane distribution analysis of the HBsAg indicated that K160R resulted in a decreased presence of the extracellular segment of HBsAg, primarily concentrated within the lipid bilayer (Fig. 6B). These alterations may potentially affect the transmembrane distribution of mutant HBsAg, impacting its secretion and leading to intracellular aggregation. As for E2G in genotype C, the hydrophilic analysis was not feasible due to its proximity to the protein terminus. Nevertheless, its location near TMD1 and the substantial change in hydrophobic value from glutamate (−3.500) to glycine (−0.400) could also influence its secretion. Moreover, the presence of the S132F mutation in genotype B led to a notable reduction in intracellular HBsAg and an increase in extracellular HBsAg, facilitating the secretion of HBsAg. This distinctive pattern differed significantly from mutations observed at the same locus, such as S132G/Y. The S132F mutation induced a substantial shift in the local site’s properties, transitioning from a hydrophilic to a more hydrophobic structure, which enhances the hydrophobic characteristics of the MHR (Fig. 6C). Additionally, S132F led to a marked elevation in the proportion of the extracellular segment of HBsAg, which showed a tendency to protrude from the membrane (Fig. 6D). Consequently, these findings provided a plausible explanation for the stimulatory impact of the S132F mutation on HBsAg secretion.

## DISCUSSION

In this study, OBI samples were reclassified with an ultrasensitive HBsAg assay. The true OBI-related mutations were analyzed in the Fujirebio− OBI group. We conducted a detailed analysis of their impact on the expression of HBsAg and their influence on the detection capability of various commonly utilized assays for HBsAg detection.

Using the Lumipulse G HBsAg-Quant assay, 45 out of 90 (50.00%) genotype B samples and 51 out of 118 (43.22%) genotype C samples tested positive for HBsAg, which were deemed negative through commonly utilized assays. These findings align with previous studies reporting detection rates ranging from 18.2% to 61.5% using more sensitive HBsAg reagents (10, 15, 21, 22). Therefore, integrating ultrasensitive HBsAg assays could significantly improve HBV diagnosis, particularly for individuals with minimal HBsAg levels, in HBV-endemic areas like the Western Pacific region, and in regions where anti-HBc is excluded from routine blood donation screening. The increased likelihood of false positive outcomes resulting from enhanced sensitivity also warrants attention and should be interpreted in conjunction with other serological results. This is expected to contribute to the goal of achieving a 90% diagnosis rate by 2030 as outlined in the global health sector strategies. Previous studies have also indicated the advantages of ultrasensitive HBsAg reagents in monitoring HBV infection post-treatment (26, 27). Our study proposes that the utilization of ultrasensitive reagents can facilitate the identification and investigation of OBI. We observed that compared to the Fujirebio+ group, the Fujirebio− OBI group exhibited lower levels of HBsAg and HBV DNA, along with higher levels of anti-HBs. Overall, ultrasensitive reagents are beneficial for the therapeutic strategies and mechanisms related to HBV.

Sequence alignment revealed single mutations in 17 sites in genotype B and 16 sites in genotype C occurred more frequently in OBI samples. Notably, our study firstly reported that mutations A5S, T123C/K/P, A128V, S132G/I/T, K141C/N/T, P142I/K/R/S/T, and S193L in genotype B, as well as F93L/S, M103I, S114P, and I226S in genotype C are associated with OBI. Among these mutations, M103I (28), T123K (29), A128V (30), K141N/T (31, 32), P142I (29), S193L (33), and I226S (34) have been previously reported in other genotypes. Additionally, nine combined mutations in genotype B and nine combined mutations in genotype C were also analyzed in this study. Multiple combined mutations in naturally infected HBV strains are common due to the high mutation rate. Combined mutations are believed to have a more significant influence on the biological activity of HBV. Unfortunately, there is a limited amount of research available that examines the combined mutations within the S region (18, 20, 35). Investigating these combined mutations may provide valuable insights into the mechanisms of OBI in future studies.

Certain mutations may modify the structure of the HBsAg antigen, leading to an alteration in the antigenic determinant and a disruption in the recognition by anti-HBs (12). The mutant HBsAg in cell supernatants were produced through a plasmid containing the whole HBV genome and subsequently assessed with various commercial reagents commonly used in clinic. In genotype B, mutations at position T123 significantly impacted the detection by DiaSorin-Murex HBsAg Version 3, Roche-HBsAg II quant, and Fujirebio-Lumipulse G HBsAg-Quant assay. The effect of T123 mutations was also evident in other genotypes (36, 37), underscoring the crucial role of this site in interacting with neutralizing antibodies. K141C affected the detection by Roche assay. Meanwhile, mutations of P142 notably affected both Roche and Abbott. In genotype C, S114A/P, T115N, T118K, P120Q, and T118K+S174N exhibited reduced detection results by the Fujirebio assay. The interference of mutations at positions T118K and P120Q has been reported to result in lower outcomes for the Fujirebio assay compared to the Abbott assay (38, 39). Noticeably, these mutations are predominantly situated in the MHR region, aligning with the detection sites of numerous reagents. In our study, these mutations that affect the detection capacity of commonly utilized reagents in clinical settings occurred in 7.29% (7/96) OBI strains of genotype B and 20.59% (28/136) of genotype C. These findings indicate the importance of evaluating the impact of mutations on the efficacy of reagents, especially for those with limited HBsAg levels (10).

Our study identified 15 single mutations and four combined mutations in genotypes B and C that led to a decrease in both extracellular and intracellular HBsAg. These mutations may impact the production or stability of HBsAg. Among them, T123C/K and D144G in genotype B and Q101H and M103I in genotype C also caused a reduction in the ratio of extracellular to intracellular HBsAg and secretion disorders (Table 2). M103I, D144G, and G145A in genotype A have been reported to cause similar results (40, 41). Consecutive mutations P142L/R/S/T, T143M, D144G, and G145A all located in the second loop of MHR significantly impact HBsAg expression. Consistent with previous studies, mutations in MHR may not only affect antigenicity but also lead to reduced HBsAg expression (42, 43). Additionally, we identified certain mutations that impede the secretion of HBsAg, elucidated through transmembrane distribution and hydrophobicity analysis. K160R in genotype B and E2G in genotype C resulted in decreased extracellular HBsAg and increased intracellular HBsAg, which were known as intracellular aggregation. Similarly, E2G in genotype B has been reported to cause intracellular accumulation and reduced secretion of HBsAg (44). Mutations, especially those causing significant changes in hydrophilic properties and located near the hydrophilic interface of the protein, may alter the membrane distribution of HBsAg and thus affect secretion. Upregulated intracellular HBsAg may lead to endoplasmic reticulum stress and mitochondrial autophagy, thus promoting hepatocellular carcinoma (45, 46). Overall, we identified and characterized some mutations in the S region that caused decreased production, stability of HBsAg, and secretion disorders. The decreased extracellular HBsAg may lead to undetectable HBsAg in OBI.

In addition, we observed that some OBI-related mutations did not lead to the expected reduction in extracellular HBsAg levels. This phenomenon may be related to the effects of combined mutations. In genotype B, the Q181R mutation caused an increase in supernatant HBsAg levels, but the frequent occurrence of the double mutation Q181R+M103I ultimately led to a decrease in supernatant HBsAg levels. The additional impact of combined mutations was also observed in Q129P+V168A and R160K+C221Y in genotype C. Additionally, different amino acid mutations at the high-frequency mutation site S132 produced varying effects. Although S132F caused an increase in extracellular HBsAg levels, this mutation only accounted for a small portion of the mutated amino acids, while mutations including S132G/Y significantly reduced extracellular HBsAg levels. In the intricate natural infection, the composition of mutations becomes more complex, and the combination of various mutations may ultimately lead to OBI. In addition, S mutations that alter the stability or antigenic properties of HBsAg may also contribute to the occurrence of OBI. These mechanisms may explain the unexpected effects of some OBI-related mutations.

There are some limitations to this research. Firstly, the cross-sectional study lacked follow-up, which could not reflect the dynamic changes of HBV during infection and replication. The studied sequences were dominant strains in blood donors, whereas natural infections are typically more complex. Secondly, our validation was preliminary and conducted *in vitro*. Differences in the cell supernatant and human serum matrix may affect the detection. Thirdly, our study focused on genotype B and genotype C, which is consistent with the distribution of the distribution of HBV genotypes in China and in East Asia (47, 48). Therefore, we lack samples of other genotypes in OBI cases globally. Fourthly, in this study, we utilized a forward validation approach based on wild-type plasmids, without conducting reverse verification through corrective experiments (49, 50). Implementing a two-way verification would enhance the robustness of the conclusions drawn.

In conclusion, our study first conducted sequence analysis on Fujirebio− OBI samples obtained from blood donors to identify OBI-related mutations. The results demonstrate that the employment of ultrasensitive HBsAg assay proved to be beneficial in re-classifying OBI infections as chronic hepatitis B with HBsAg-positivity. We also further delineated the impact of mutations on the expression and antigenicity of HBsAg, providing a preliminary analysis for potential underlying mechanisms. Generally, our study offers a new perspective on OBI-related mutations by ultrasensitive HBsAg assay and lays the groundwork for further research on the OBI mechanism and the enhancement of HBsAg detection reagents.

## Data Availability

Data will be made available on request.

## SUPPLEMENTAL MATERIAL

Supplemental material jcm.02071-24-s0001.docx Tables S1 to S3; Fig. S1 to S3.

ASM does not own the copyrights to Supplemental Material that may be linked to, or accessed through, an article. The authors have granted ASM a non-exclusive, world-wide license to publish the Supplemental Material files. Please contact the corresponding author directly for reuse.
