# Supplementary material for: Identification and characteristics of mutations promoting occult HBV infection by ultrasensitive HBsAg assay
Source: J Clin Microbiol. 2025 Mar 31;63(5):e02071-24. doi: 10.1128/jcm.02071-24 (PMC12077177; doi:10.1128/jcm.02071-24)
Supplement: Supplemental material — Tables S1 to S3; Fig. S1 to S3. [file jcm.02071-24-s0001.docx]

**Supplementary materials**

**Identification and Characteristics of Mutations Promoting Occult HBV Infection by Ultrasensitive HBsAg assay**

Shi Song, Qian Su, Ying Yan, Huimin Ji, Huizhen Sun, Kaihao Feng, Abudulimutailipu Nuermaimaiti, Shana Halemubieke, Ling Mei; Xinru Liu, Zhuoqun Lu, Le Chang, Lunan Wang

**Supplementary Table 1. Amplification primers for preS, S, and C regions of HBV strains**

| Primer name^+^ | | Primer sequences (5’-3’) |
| --- | --- | --- |
| S region | | |
| First PCR | HBV-56F | CCTGCTGGTGGCTCCAGTTC |
|  | HBV-1253R | GCAGTATGGATCGGCAGAGGAG |
| Second PCR^++^ | HBV-178F | CCTAGGACCCCTGCTCGTGTTACAGGC |
|  | HBV 1186R | CCAGTGGGGGTTGCRTCAGC |
| preS region | | |
| First PCR | HBV-2804F | GCCTCATTTTGYGGGTCACCAT |
|  | HBV-668R | CTGAGGCCCACTCCCATAG |
| Second PCR^++^ | HBV-2833F | GGGAACAAGAGCTACAGCATGG |
|  | HBV-309R | GGTTGGGGACTGCGAATTT |
| C region | | |
| First PCR | HBV-1610F | ATGGAGACCACCGTGAACG |
|  | HBV-2488R | GTAAAGTTTCCCACCTTATGAGTCC |
| Second PCR^++^ | HBV-1610F | ATGGAGACCACCGTGAACG |
|  | HBV-2465R | TATGAGTCCAAGGAATACTAACATTG |

^+^The primers were named with location in 3215bp-HBV genome of genotype B and C. The suffix F after the primer names stands for Forward primer, and suffix R stands for Reverse primer.

^++^The primers of second PCR were used for sanger sequencing.

**Supplementary Table 2. Site-directed mutagenesis primers for HBV**

| Primer name | Primer sequences (5’-3’) |
| --- | --- |
| Genotype B - Single mutations | |
| GTB-A5T-F1 | GGTCCTAGGAGTCCTGACGTGATGTTCTCCATGTTCG |
| GTB-A5T-R1 | CGAACATGGAGAACATCACGTCAGGACTCCTAGGACC |
| GTB-A5S-F1 | TCCTAGGAGTCCTGATGAGATGTTCTCCATGTTCG |
| GTB-A5S-R1 | CGAACATGGAGAACATCTCATCAGGACTCCTAGGA |
| GTB-S114T-F1 | CGGTGCTGGTGGTCGTTGATCCTGGAATTAGAGGACAAACG |
| GTB-S114T-R1 | CGTTTGTCCTCTAATTCCAGGATCAACGACCACCAGCACCG |
| GTB-P120Q-F1 | GTGCAGGTTTTGCATTGTCCGGTGCTGGTGG |
| GTB-P120Q-R1 | CCACCAGCACCGGACAATGCAAAACCTGCAC |
| GTB-K122R-F1 | GAGTCGTGCAGGTTCTGCATGGTCCGGTG |
| GTB-K122R-R1 | CACCGGACCATGCAGAACCTGCACGACTC |
| GTB-P127T-F1 | GAGGTTCCTTGAGCAGTAGTCGTGCAGGTTTTG |
| GTB-P127T-R1 | CAAAACCTGCACGACTACTGCTCAAGGAACCTC |
| GTB-A128V-F1 | CATAGAGGTTCCTTGAACAGGAGTCGTGCAGGT |
| GTB-A128V-R1 | ACCTGCACGACTCCTGTTCAAGGAACCTCTATG |
| GTB-Q129R-F1 | AAACATAGAGGTTCCTCTAGCAGGAGTCGTGCAGGTTTTGC |
| GTB-Q129R-R1 | GCAAAACCTGCACGACTCCTGCTAGAGGAACCTCTATGTTT |
| GTB-T143M-F1 | GCAGTTTCCGTCCATAGGTTTTGTACAGCAACATGAGG |
| GTB-T143M-R1 | CCTCATGTTGCTGTACAAAACCTATGGACGGAAACTGC |
| GTB-D144G-F1 | TACAGGTGCAGTTTCCGCCCGTAGGTTTTGTACAG |
| GTB-D144G-R1 | CTGTACAAAACCTACGGGCGGAAACTGCACCTGTA |
| GTB-G145A-F1 | GAATACAGGTGCAGTTTGCGTCCGTAGGTTTTGTA |
| GTB-G145A-R1 | TACAAAACCTACGGACGCAAACTGCACCTGTATTC |
| GTB-K160R-F1 | CCCACTCCCATAGGTATCTTGCGAAAGCCCAAGAT |
| GTB-K160R-R1 | ATCTTGGGCTTTCGCAAGATACCTATGGGAGTGGG |
| GTB-K160N-F1 | CCCACTCCCATAGGTAATTTGCGAAAGCCCAAGAT |
| GTB-K160N-R1 | ATCTTGGGCTTTCGCAAATTACCTATGGGAGTGGG |
| GTB-Q181R-F1 | AAGCCCTACGAACCACCGAACAAACGGCACTAG |
| GTB-Q181R-R1 | CTAGTGCCGTTTGTTCGGTGGTTCGTAGGGCTT |
| GTB-S193L-F1 | ATCATCCATATAACTAAAAGCCAGACAGTGGGGGAAAG |
| GTB-S193L-R1 | CTTTCCCCCACTGTCTGGCTTTTAGTTATATGGATGAT |
| Genotype B - Combined mutations | |
| GTB-L175S-S174N-F1 | CAAACGGCACTAGTGAATTGAGCCAAGAGAAACGG |
| GTB-L175S-S174N-R1 | CCGTTTCTCTTGGCTCAATTCACTAGTGCCGTTTG |
| GTB-Q181R-V177A-F1 | CCACCGAACAAACGGCGCTAGTAAACTGAGCCA |
| GTB-Q181R-V177A-R1 | TGGCTCAGTTTACTAGCGCCGTTTGTTCGGTGG |
| GTB-I4T-F1 | GTCCTGATGCGGTGTTCTCCATGTTCGGTACAG |
| GTB-I4T-R1 | CTGTACCGAACATGGAGAACACCGCATCAGGAC |
| GTB-M103I-F1 | GACAAACGGGCAATATACCTTGATAGTCCAGAAGAACC |
| GTB-M103I-R1 | GGTTCTTCTGGACTATCAAGGTATATTGCCCGTTTGTC |
| GTB-V168A-F1 | GCCAAGAGAAACGGGCTGAGGCCCACTCC |
| GTB-V168A-R1 | GGAGTGGGCCTCAGCCCGTTTCTCTTGGC |
| GTB-S174N-F1 | CAAACGGCACTAGTAAATTGAGCCAAGAGAAACGG |
| GTB-S174N-R1 | CCGTTTCTCTTGGCTCAATTTACTAGTGCCGTTTG |
| GTB-L175S-F1 | AACAAACGGCACTAGTGAACTGAGCCAAGAGAAACG |
| GTB-L175S-R1 | CGTTTCTCTTGGCTCAGTTCACTAGTGCCGTTTGTT |
| GTB-V177A-F1 | ACCACTGAACAAACGGCGCTAGTAAACTGAGCCAA |
| GTB-V177A-R1 | TTGGCTCAGTTTACTAGCGCCGTTTGTTCAGTGGT |
| GTB-P217L-F1 | CCCAAAGACAAAAGAAAATTAGTAACAGCGGCATAAAGGGA |
| GTB-P217L-R1 | TCCCTTTATGCCGCTGTTACTAATTTTCTTTTGTCTTTGGG |
| Genotype C - Single mutations | |
| GTC-E2G-F1 | CCTGATGTTGTGCTCCCCATGTTCGGTGCAG |
| GTC-E2G-R1 | CTGCACCGAACATGGGGAGCACAACATCAGG |
| GTC-Q101K-F1 | AACGGGCAACATACCTTTGTAGTCCAGAAGAACCA |
| GTC-Q101K-R1 | TGGTTCTTCTGGACTACAAAGGTATGTTGCCCGTT |
| GTC-Q101H-F1 | CGGGCAACATACCATGGTAGTCCAGAAGAACC |
| GTC-Q101H-R1 | GGTTCTTCTGGACTACCATGGTATGTTGCCCG |
| GTC-M103I-F1 | GACAAACGGGCAATATACCTTGGTAGTCCAGAAGAACC |
| GTC-M103I-R1 | GGTTCTTCTGGACTACCAAGGTATATTGCCCGTTTGTC |
| GTC-S114A-F1 | CCCGTGCTGGTAGTTGCTGTTCCTGGAAGTAGA |
| GTC-S114A-R1 | TCTACTTCCAGGAACAGCAACTACCAGCACGGG |
| GTC-S114P-F1 | CCCGTGCTGGTAGTTGGTGTTCCTGGAAGTAGA |
| GTC-S114P-R1 | TCTACTTCCAGGAACACCAACTACCAGCACGGG |
| GTC-T115N-F1 | CCCGTGCTGGTATTTGATGTTCCTGGAAGTAGAGG |
| GTC-T115N-R1 | CCTCTACTTCCAGGAACATCAAATACCAGCACGGG |
| GTC-T116A-F1 | TCCCGTGCTGGCAGTTGATGTTCCTGGAAGTAG |
| GTC-T116A-R1 | CTACTTCCAGGAACATCAACTGCCAGCACGGGA |
| GTC-T116S-F1 | GTCCCGTGCTGCTAGTTGATGTTCCTGGAAGT |
| GTC-T116S-R1 | ACTTCCAGGAACATCAACTAGCAGCACGGGAC |
| GTC-P120Q-F1 | GTGCAGGTCTTGCATTGTCCCGTGCTGGTAG |
| GTC-P120Q-R1 | CTACCAGCACGGGACAATGCAAGACCTGCAC |
| GTC-P120T-F1 | GTGCAGGTCTTGCACGTTCCCGTGCTGGTAGTTGA |
| GTC-P120T-R1 | TCAACTACCAGCACGGGAACGTGCAAGACCTGCAC |
| GTC-K122R-F1 | GAATCGTGCAGGTCCTGCATGGTCCCGTG |
| GTC-K122R-R1 | CACGGGACCATGCAGGACCTGCACGATTC |
| GTC-E164G-F1 | CGGACTGAGGCCCACCCCCATAGGAATCTTG |
| GTC-E164G-R1 | CAAGATTCCTATGGGGGTGGGCCTCAGTCCG |
| GTC-S167L-F1 | AGGAGAAACGGACTAAGGCCCACTCCCATAG |
| GTC-S167L-R1 | CTATGGGAGTGGGCCTTAGTCCGTTTCTCCT |
| GTC-V168A-F1 | CCAGGAGAAACGGGCTGAGGCCCACTC |
| GTC-V168A-R1 | GAGTGGGCCTCAGCCCGTTTCTCCTGG |
| GTC-L175S-F1 | AACAAATGGCACTAGTGAACTGAGCCAGGAGAAACG |
| GTC-L175S-R1 | CGTTTCTCCTGGCTCAGTTCACTAGTGCCATTTGTT |
| GTC-V177A-F1 | ACCACTGAACAAATGGCGCTAGTAAACTGAGCCAG |
| GTC-V177A-R1 | CTGGCTCAGTTTACTAGCGCCATTTGTTCAGTGGT |
| GTC-Q181R-F1 | AAAGCCCTACGAACCACCGAACAAATGGCACTAGT |
| GTC-Q181R-R1 | ACTAGTGCCATTTGTTCGGTGGTTCGTAGGGCTTT |
| GTC-I226S-F1 | GTTTGGTTTTATTGGGGTTCAACTGTATACCCAAAGACAAAAGAAAA |
| GTC-I226S-R1 | TTTTCTTTTGTCTTTGGGTATACAGTTGAACCCCAATAAAACCAAAC |
| Genotype C- Combined mutations | |
| GTC-T118K-F1 | CTTGCATGGTCCCTTGCTGGTAGTTGATGTTCCTG |
| GTC-T118K-R1 | CAGGAACATCAACTACCAGCAAGGGACCATGCAAG |
| GTC-Q129P-F1 | GAAACATAGAGGTTCCTGGAGCAGGAATCGTGCAG |
| GTC-Q129P-R1 | CTGCACGATTCCTGCTCCAGGAACCTCTATGTTTC |
| GTC-R160K-F1 | CCACTCCCATAGGAATTTTGCGAAAGCCCAGGA |
| GTC-R160K-R1 | TCCTGGGCTTTCGCAAAATTCCTATGGGAGTGG |
| GTC-S174N-F1 | CAAATGGCACTAGTAAATTGAGCCAGGAGAAACGG |
| GTC-S174N-R1 | CCGTTTCTCCTGGCTCAATTTACTAGTGCCATTTG |
| GTC-C221Y-F1 | CAAATGTATACCCAAAGATAAAAGAAAATTGGTAATAGAGGTAAAAAGGGACT |
| GTC-C221Y-R1 | AGTCCCTTTTTACCTCTATTACCAATTTTCTTTTATCTTTGGGTATACATTTG |

Note: the suffix F1 after the primer names stands for Forward primer, and suffix R1 stands for Reverse primer.

**Supplementary Table 3. Number of obtained sequences in preS region, S region and C region in OBI group and HBsAg+ group**

|  | OBI group  n=238 | | | HBsAg+ group  n=538 | | |
| --- | --- | --- | --- | --- | --- | --- |
|  | preS | S | C | preS | S | C |
| genotype B | 93 | 96 | 82 | 170 | 353 | 170 |
| genotype C | 123 | 136 | 123 | 132 | 167 | 112 |

**Supplementary Figure 1**


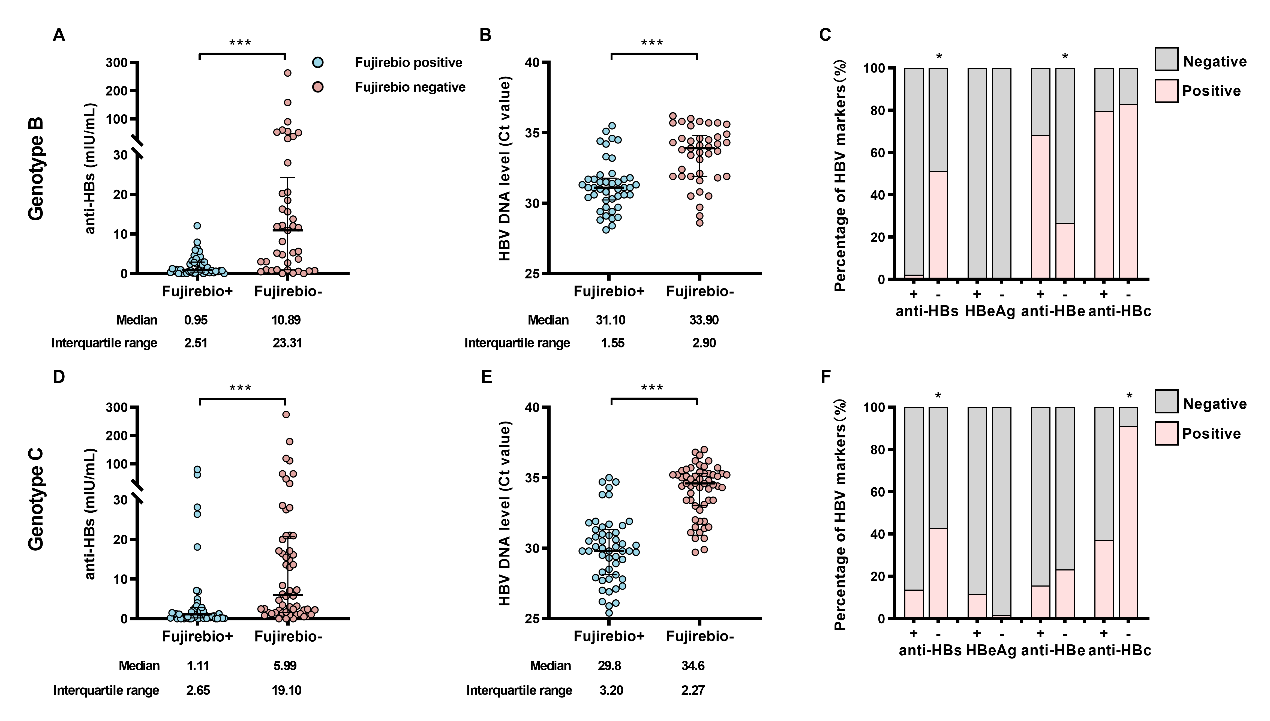


**Supplementary Figure 1. Serological characteristics of Fujirebio+ OBI and Fujirebio- OBI groups in genotype B and genotype C.** Serum level of anti-HBs(A) and HBV DNA (B) of genotype B samples. (C)Positive percentage of HBV markers including anti-HBs, HBeAg, anti-HBe, and anti-HBc in genotype B samples. Serum anti-HBs (F) and HBV DNA (G) of genotype C samples. (H)Positive percentage of HBV markers including anti-HBs, HBeAg, anti-HBe and anti-HBc in genotype C samples. The corresponding median and P value between Fujirebio+ and Fujirebio- OBI were shown. Results were analyzed by normality test and Mann-Whitney U test, *P<0.05, **P<0.01, ***P<0.001.

**Supplementary Figure 2**


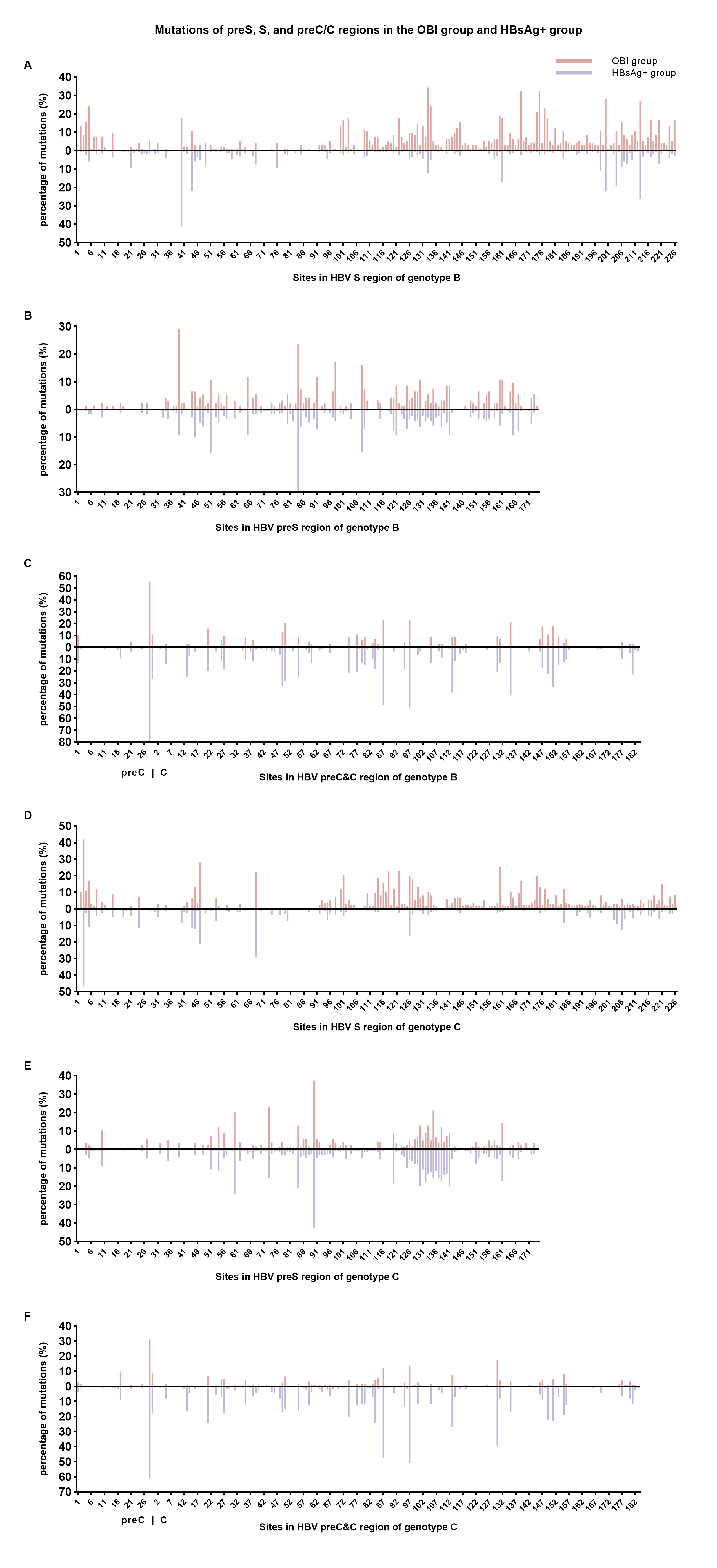


**Supplementary Figure 2. Mutations of preS, S, and preC/C regions in the OBI group and HBsAg+ group.** The comparison of mutation patterns in the S (A), preS (B), and preC/C (C) regions in genotype B between the OBI group and the HBsAg+ group. The comparison of mutation patterns in the S (D), preS (E), and preC/C (F) regions in genotype B between the OBI group and the HBsAg+ group. The mutation rates in the OBI group and the HBsAg+ group are presented on the upper and lower parts of the y-axis, respectively, marked in brown and purple.

**Supplementary Figure 3**
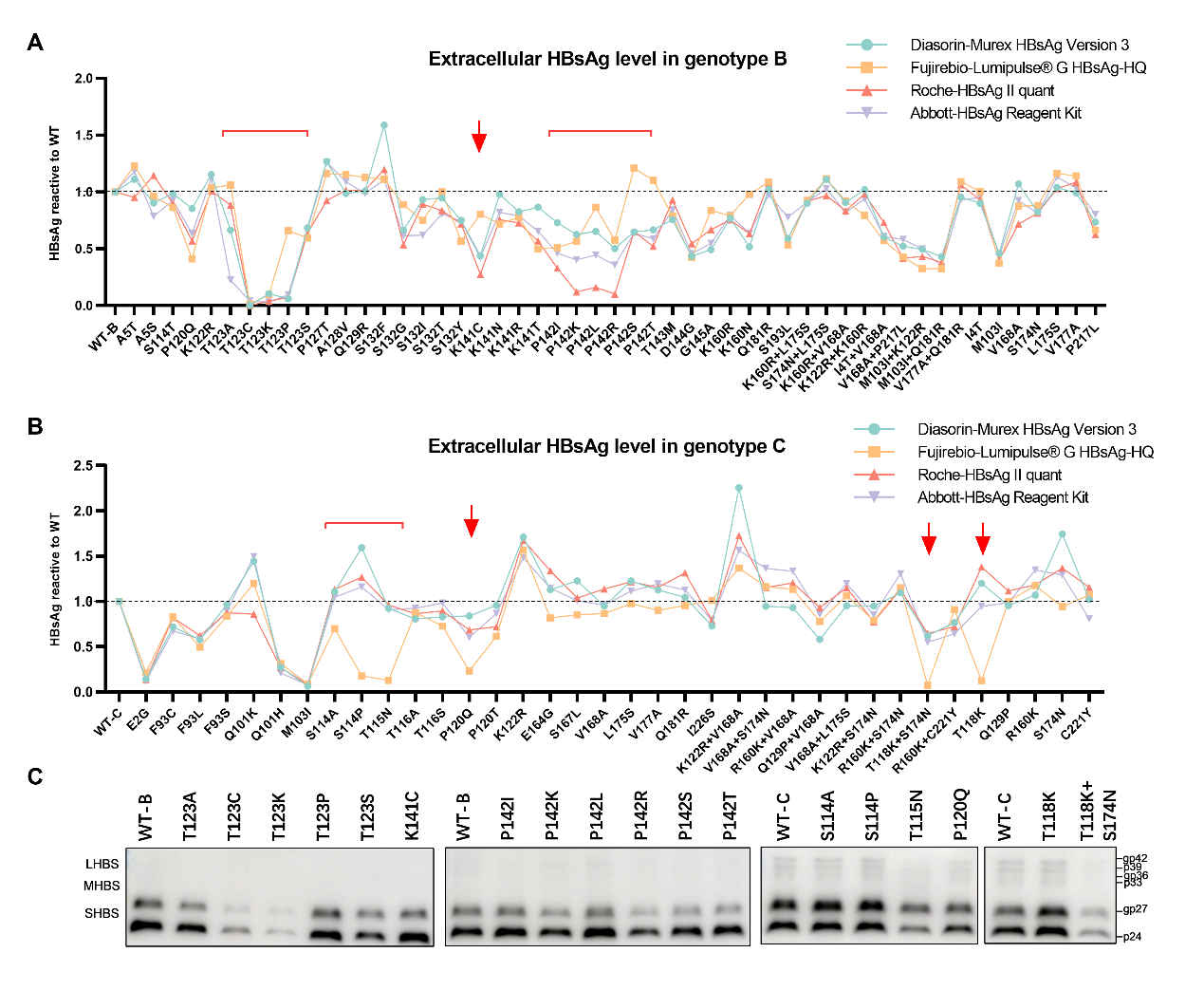


**Supplementary Figure 3. Extracellular HBsAg analysis with different detection methods.** The supernatants were collected 72h after transfection with WT or mutant plasmids. (A and B) Extracellular HBsAg in the same batch of supernatants of genotype B/C were detected by DiaSorin, Abbott, Roche, and Fujirebio. Results were expressed as a relative value compared to the corresponding WT. The mutant samples were displayed as single mutations, combined mutations and complementary mutations for combined mutations. Certain mutations that led to intricate outcomes were highlighted by red lines or arrows. (C) Extracellular HBsAg of partially mutations were detected by Western Blot for confirmation. SHBS, MHBS and LHBS stand for small, middle and large HBsAg respectively.
